# Association between serum irisin concentration and ischemic stroke: From etiology to clinic

**DOI:** 10.5937/jomb0-36681

**Published:** 2022-10-15

**Authors:** Mustafa Çalık, Yildizhan Sengul, Gurkan Zahide Mail, Deniz Hintoglu, Mısırlıoglu Naile Fevziye, Hafize Uzun

**Affiliations:** 1 University of Health Sciences, Gaziosmanpa a Training and Research Hospital, Department of Emergency Medicine, İstanbul, Turkey; 2 University of Health Sciences, Gaziosmanpasa Training and Research Hospital, Department of Neurology, Istanbul, Turkey; 3 University of Health Sciences, Gaziosmanpa a Training and Research Hospital, Department of Biochemistry, Istanbul, Turkey; 4 Istanbul Atlas University, Faculty of Medicine, Department of Medical Biochemistry, Istanbul, Turkey

**Keywords:** acute ischemic stroke, irisin, National Institutes of Health Stroke Scale, aetiology, akutni ishemijski moždani udar, irisin, skala moždanog udara Nacionalnog instituta za zdravlje, etiologija

## Abstract

**Background:**

To investigate the relationship between irisin levels in serum and classification of subtype of acute ischemic stroke, National Institutes of Health Stroke Scale (NIHSS) and Modified Rankin Score (mRS) at the time of discharge from the hospital in Turkish patients who had their first acute ischemic stroke (AIS).

**Methods:**

Serum irisin levels were measured using enzyme linked immunosorbent assay (ELISA) 180 patients who applied to emergency department with the diagnosis of AIS from May 2021 to November 2021.

**Results:**

A significant relationship was found between serum irisin levels and ischemic stroke aetiological factors (TAOST) (p=0.017). Increased serum irisin levels were detected in patients without neurological deficits with localization value than those with it (p<0.01). Serum irisin levels also have a negative correlation with high-density lipoprotein (HDL) value in ischemic stroke (r: -0.272, p<0.01).

**Conclusions:**

High serum irisin levels found in patients with stroke attributed to small vessel disease and in patients with ischemic stroke in whom we did not find any neurological deficits with a localization value. The results of the study show that serum irisin levels have an important role in the etiology of ischemic stroke. Although the question how the irisin is involved in the course of ischemic stroke and what the clinical reflection has not been answered, these findings are a pioneering study on this subject.

## Abstract (sr)

**Uvod:**

Cilj je bio da se istraži odnos između nivoa irisina u serumu i klasifikacije podtipa akutnog ishemijskog moždanog udara, skale Nacionalnog instituta za zdravstveni moždani udar (NIHSS) i modifikovanog Rankinovog skora (mRS) u tre nutku otpuštanja iz bolnice kod turskih pacijenata koji su imali prvi akutni ishemijski moždani udar (AIS).

**Metode:**

Nivo irisina u serumu meren je enzimskim imuno-sorbentnim testom (ELISA) kod 180 pacijenata koji su se pri javili u hitnu pomoć sa dijagnozom AIS od maja 2021. do novembra 2021.

**Rezultati:**

Utvrđena je značajna veza između nivoa irisina u serumu i etioloških faktora ishemijskog moždanog udara. (TAOST) (p=0,017). Povećani nivoi irisina u serumu otkriveni su kod pacijenata bez neuroloških deficita sa vrednostima pri lokalizaciji od onih sa njim (p<0,01). Nivoi irisina u serumu takođe imaju negativnu korelaciju sa vrednošću lipoproteina visoke gustine (HDL) kod ishemijskog moždanog udara (r: -0,272, p<0,01).

**Zaključak:**

Visok nivo irisina u serumu utvrđen je kod pacijenata sa moždanim udarom koji se pripisuje bolesti malih krvnih sudova i kod pacijenata sa ishemijskim moždanim udarom kod kojih nismo pronašli neurološke deficite sa lokalizacionom vrednošću. Rezultati studije pokazuju da nivoi irisina u serumu imaju važnu ulogu u etiologiji ishemijskog moždanog udara. Iako nije odgovoreno na pitanje kako je irisin uključen u tok ishemijskog moždanog udara i šta je klinika refleksija, ovi nalazi su pionirska studija na ovu temu.

## Introduction

Stroke is a medical emergency caused by the interrupted blood supply to the brain that further leads to rapid loss of brain functions. It is the second leading cause of death worldwide and is associated with long-term disability [1]. Especially in low- and middle-income countries, the incidence of stroke-related mortality is increasing, resulting in a high economic burden for both the patients and society [2]. Ischemic stroke accounts for ~80% of stroke cases [3].

Irisin is a small polypeptide hormone that is cleaved from the fibronectin type III domain containing 5 (FNDC5) [4]. Recently, several studies have confir med that irisin plays a protective role in the pathogenesis of many diseases, including neurodegenerative diseases such as Alzheimer's disease, and cardiovascular diseases [5] [6] [7]. Furthermore, a previous study showed that low serum irisin levels were a predictor of poor early functional outcome in ischemic stroke patients [8]. Another study has found that plasma irisin also decreases after ischemic stroke which suggests that the release of irisin from muscles into the blood is inhibited after ischemic stroke [9]. How the irisin is involved in the course of ischemic stroke and what the clinical reflection of this are still unknown and needs to be clarified.

In this study, the relationship between serum irisin levels in serum and classification of subtype of acute ischemic stroke (Trial of Org 10172 in Acute Stroke Treatment, TOAST), National Institutes of Health Stroke Scale (NIHSS) and Modified Rankin Score (mRS) at the time of discharge from the hospital in Turkish patients who had their first acute ischemic stroke (AIS) were investigated.

## Materials and methods

This study was conducted at 180 patients who applied to emergency department with the diagnosis of AIS from May 2021 to November 2021. The protocol for sample collection was approved by the Local Training and Research Hospital's Ethical Committee (ethical approval number: 285/05.2021). The study was performed in accordance with the Helsinki Declaration. All participants or their relatives were informed of the study protocol, and their written informed consents were obtained.

### The inclusion criteria

Patients older than 18 years of age admitted to the emergency department with AIS (acute cerebrovascular events in which ischemia was demonstrated in CT scan and brain MRI in the first 24 hours of their arrival at the emergency department).

### The exclusion criteria

(1) Under 18 years old, (2) Intracerebral haemorrhage and other neurological disease (different from acute ischemic stroke), (3) Malignancy, (4) Those with liver and kidney failure, (5) Those who abuse alcohol and drugs, (6) Those diagnosed with autoimmune or rheumatological diseases, (7) Those who use metronidazole or warfarin (Other anticoagulants), (8) history of any kind of trauma and (9) Patients with acute of subacute infection such as COVID-19, (10) Patients who did not give informed consent/did not want to participate in the study.

Twenty-three patients who did not give informed consent / did not want to participate in the study, 31 patients diagnosed with haemorrhagic stroke, 1 patient admitted due to trauma, 8 patients using anticoagulants, 9 patients diagnosed with cancer, 4 patients diagnosed with myocardial infarction, 6 patients with chronic renal failure, 3 patients who has head trauma and 5 patients who had acute infection were excluded from the study. Ninety patients who were diagnosed with ischemic stroke based on computerized tomography (CT) and magnetic resonance imaging (MRI) after excluding the patients from our study according to the above mentioned exclusion criteria were included in the study.

Demographic characteristics, risk factors (all medical history), habits and vital signs of the patients were recorded in the hospital information system. CT, MRI, CT angiography, carotid doppler ultrasonography, echocardiography and 24-hour-Holter-ECG were performed in each patient.

Strokes were classified according to the criteria of the TOAST (Trial of Org 10172 in Acute Stroke Treatment) classification. The TOAST classification denotes five subtypes of ischemic stroke: 1) large-artery atherosclerosis, 2) cardioembolism, 3) small-vessel occlusion, 4) stroke of other determined aetiology, and 5) stroke of undetermined aetiology. Patients were evaluated with the National Institute of Health Stroke Scale (NIHSS). NIHSS, was calculated both at the first admission of the patients to the emergency department, and at discharge from the hospital. Those with an NIHSS value greater than 5 were considered major stroke, and those with a NIHSS value of 5 or less were considered as minor strokes (at the first admission). Functional outcome was also obtained according to the modified Rankin Scale (mRS) score. Those with mRS scores of 0 to 2 were classified as well-functioning, and those with scores of 3 to 6 were classified as poorly functioning. Patients were subdivided into two groups: (1) Patients who had neurological deficits with localization value (2) Patients who did not have neurological deficits with localization value.

Venous blood was drawn from each patient in the biochemistry tube within the first 24 hours of the emergency department admission. Serum samples were obtained after at least 30 min of clotting by centrifugation at 2.500 g for 15 min and were stored at-80 C until assayed for determination of irisin concentrations. All icteric or haemolysed blood sample were discarded. All parameters were analysed in all samples together in a single batch; after we had finished our protocol (control and patient samples were analysed in the same batch).

### Measurement of serum irisin concentration

Serum irisin concentration were in duplicates analysed by a commercially available competitive enzyme linked immunoassay kit (Human Irisin ELISA kit, Cat no: E3253Hu, Bioassay Technology Laboratory, Nanhu Dist, Jiaxing, Zhejiang, China). The coefficients of intra and inter assay variation were 8% (n = 25) and 10% (n = 25), respectively.

Glucose and lipid profiles were measured with an autoanalyzer (COBAS 8000, ROCHE-2007, Tokyo, Japan).

CRP analysed using a nephelometric method with an autoanalyzer (Immage 800 Beckman Coulter).

Activated partial prothrombin time (APTT) and prothrombin time (PT) as parameters of blood compatibility were measured with an automatic coagulation analyser (STAR Evolution, Diagnostica Stago, Assiernes, France).

### Statistical analysis

All statistical analyses were performed using SPSS 22.0 (SPSS Inc., Chicago, IL, USA). The parametric variables were expressed as mean±standard deviation (SD). The normal distribution of each continuous variable was assessed using Kolmogorov-Smirnov tests. Mann-Whitney tests were used in two group comparisons, Kruskal Wallis test was used for comparison of more than two groups. Spearman correlation analysis was used to evaluate relationship between variables. The results were considered statistically significant at p<0.05.

## Results

Ninety patients were included in the study. Mean ± SD age was 65.3 ± 12.3 years, 47.8% (n=43) of the patients were male and 52.2% (n=47) of the patients were female. Body mass index (BMI) was 25.89±5.18. The median NIHSS score on admission was 5 with interquartile range (IQR) being between 1-40. The lesion locations detected by radiological imaging methods (MRI, CT) were most common in middle cerebral artery (MCA) (n=34, 37.7%). Then, in order of frequency, they were listed as posterior cerebral artery (PCA), anterior cerebral artery (ACA), lenticulostriate artery (LSA). The general characteristics of patients presenting with ischemic stroke are described in Table 1.

**Table 1:** General characteristics of stroke patients. IQR, interquartile range; NIHSS, National Institutes of Health Stroke Scale; SBP, systolic blood pressure; DBP, diastolic blood pressure; HR, heart rate; CRP, C-reactive protein; BMI, body mass index; HDL, high-density lipoprotein; LDL, low-density lipoprotein; TG, triglyceride; TC, total cholesterol; PT, prothrombin time; aPTT, activated partial thromboplastin time.

Variable	n=90
Female, N (%)	47 (52.2)
Age, years, medians (IQR)	66 (27–91)
BMI, kg/m^2^, medians (IQR)	25.89 (20.11–30.49)
Vital parameters<br>SBP mmHg<br>DBP mmHg<br>HR /minute	<br>140 (85–220)<br>85 (48–120)<br>82 (60–102)
Vasculer risk factors, N (%)<br>Hypertension<br>Diabetes<br>Hyperlipidemia<br>Coronary Artery Disease<br>Smoking	<br>55 (61.1)<br>38 (42.2)<br>6 (6.7)<br>15 (16.7)<br>30 (33.3)
NIHSS at admission, medians (IQR)	5 (1–40)
mRS, median (IQR)	2 (0–6)
NIHSS-Exıt, median (IQR)	2 (0–24)
TAOST, N (%)<br>Large-artery atherosclerosis<br>Cardioembolism<br>Small-vessel occlusion<br>Stroke of other determined aetiology<br>Stroke of undetermined aetiology.	<br>27 (30)<br>22 (24.4)<br>9 (10)<br>26 (28.9)<br>6 (6.7)
Laboratory findings, medians (IQR)<br>TG (mmol/L)<br>TC (mmol/L)<br>HDL (mmol/L)<br>LDL (mmol/L)<br>Glucose (mmol/L)<br>CRP (mg/L)<br>PT (sec.)<br>aPTT (sec.)<br>Irisin (mg/L)	<br>1.71 (0.38–8.10)<br>5.05 (2.92–14.43)<br>1.05 (0.54–1.79)<br>3.17 (1.06–10.36)<br>6.49 (4.10–21.81)<br>71.80 (5.80–2974)<br>13.95 (10.2–21.5)<br>28.9 (20.2–56.4)<br>5.93 (2.65–60.0)

A significant relationship was found between serum irisin levels and ischemic stroke aetiological factors (TAOST) (p=0.017) Table 2.

**Table 2:** Parameters correlated with serum irisin levels. TOAST, Trial of Org 10172 in Acute Stroke Treatment; HDL, high density lipoprotein.

Variable	Subgroup	N (%)	Mean Rank	r	p
Neurological deficits with a localization	(0=No)<br>(1=Yes)	24 (26.7)<br>66 (73.3)	62.50<br>39.32	-0.395	<0.01
TOAST	1 - Large-artery atherosclerosis<br>2 - Cardioembolism<br>3 - Small-vessel occlusion<br>4 - Stroke of other determined aetiology<br>5 - Stroke of undetermined etiology	27 (30)<br>22 (24.2)<br>9 (10)<br>26 (28.9)<br>6 (6.7)	44.87<br>47.14<br>67.89<br>35.52<br>52.00	-0.053	<0.01
HDL	*	90	*	-0.272	<0.01

**Figure 1:**
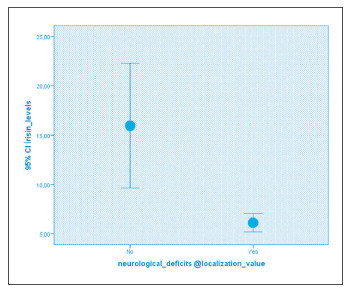
The relationship between neurological deficits with localization value in ischemic stroke and irisin levels.

Higher serum irisin levels were detected in patients without neurological deficits with localization value than those with it (p<0.01). Its relation with irisin levels is shown in Figure 1 as a subgroup.

Serum irisin levels also have a negative correlation with high-density lipoprotein (HDL) value in ischemic stroke (r: -0.272, p<0.01). However, no significant relationship was found between serum irisin levels and age, gender, BMI, mRS, NIHSS scores, vascular risk factors, smoking and outcome parameters. Moreover, no correlation was found between biochemical parameters (except HDL) and irisin levels.

## Discussion

In current study, a significant relationship was found between the etiological subtypes determined using TOAST criteria and serum irisin levels. The serum irisin levels of patients with stroke caused by small vessel disease small were found to be higher than patients with stroke caused by large vessel disease. The serum irisin levels of patients with important localization of ischemic stroke were found to be higher than those without any neurological deficit. The results of the study show that serum irisin levels have an important role in the aetiology of ischemic stroke.

A limited number of studies have investigated the relationship between irisin and stroke. Among these, an animal study by Liu et al. [10] has shown that plasma irisin levels are negatively correlated with infarct volume, neurological deficit, plasma TNF-α and plasma IL-6 concentration. The TOAST classification is a useful classification for distinguishing stroke subtypes. It is a classification based mainly on aetiology, with large vessel atherosclerosis, cardioembolic stroke, and small vessel disease of unknown and unidentified cause. Small vessel disease is a subgroup that affects small vessels with infarct volume not exceeding 15 mm. The small infarct volume may explain its correlation with irisin. Similar to the animal study, this correlation was also found in those without neurological deficits with localization value. However, in this study, although irisin levels did not provide an idea for the prognosis of stroke in patients with shortterm follow-up, further evidence is needed to investigate irisin in this regard.

Rapid identification of the ischemic stroke subtype is important both for the physician's approach and for the organization of clinical stroke studies. It is valuable in that it is the first study in the literature to examine the relationship between ischemic stroke etiological criteria (TAOST) and the relationship between neurological deficits with localization value and serum irisin levels. Furthermore, we found a negative correlation between serum irisin levels and HDL cholesterol. In current study, the serum irisin level varied from 2.65 to 60.0 μg/L. Interestingly, irisin levels in the different populations had a very large range, from 0.58 to 457.2 μg/L (10, 11). We did not find a relationship between age, gender, BMI, mRS, NIHSS, vascular risk factors, smoking and biochemical parameters and irisin levels. Recent studies have shown that age and gender are not associated with serum irisin levels. Contrary to expectations, studies have shown that serum irisin levels increase with increasing age in patients with myocardial infarction [9] [11] [12] [13] [14]. In the meta-analysis study, four articles observed lower irisin levels in obese subjects than healthy controls, while nine articles observed the opposite result, exhibiting significantly higher irisin levels in obese subjects compared to healthy controls [15]. Liu et al. [10] reported in experimental study, that compared with the middle cerebral artery occlusion (MCAO) group, the electroacupuncture (EA) group showed better behavioural performance, a smaller cerebral infarct volume, more surviving neurons, and a significant increase in peri-infarction cortex and serum of irisin levels in rats. Previous studies have shown that mRS and NIHHS are negatively correlated with irisin levels [8]. In our study, even though small differences can be observed there was no statistically significant differences between groups according to outcome and no correlation was found between irisin levels and these stroke scales. We think that the release of irisin from the muscles into the blood is inhibited as the reason why irisin levels are found to be lower in patients with AIS. According to our exclusion criteria, for some accompanying conditions or diseases, we only took a history from the patient or some situations history was taken from patient’s relatives. Another reason might be that the present study was run in the time of COVID-19 pandemic. Some patients might avoid to admit to hospital because of their fear or anxiety about the pandemic. Likewise, serum irisin is measured by ELISA, however, it varies greatly between kits. These differences are due to the diversity of irisin epitopes targeted for measurement by manufacturing companies. If the blood samples taken for irisin and other parameters in AIS were not taken at the first application, but a few days after the full establishment of the disease findings, more meaningful results could be obtained. The mechanism by which irisin mediates the neurological effect on stroke is still unknown. Serum irisin is a novel, independent prognostic marker improving currently used risk stratification of stroke patients [8] [16] [17].

According to this expletory study, increased serum irisin levels found in patients with stroke attributed to small vessel disease and in patients with ischemic stroke in whom we did not find any neurological deficits with a localization value. Although the question how the irisin is involved in the course of ischemic stroke and what the clinical reflection has not been answered, the findings are a pioneering study on this subject. Future studies with a larger patient population comparing healthy control subjects is needed to prove the accuracy of our findings and to add more information.

## Dodatak

### Acknowledgments

I would like to thank all the emergency department and neurology doctors for helping us collect serum irisin blood samples.

### Conflict of interest statement

All the authors declare that they have no conflict of interest in this work.

## References

[1] Mathers C D, Boerma T, Ma F D (2009). Global and regional causes of death. Br Med Bull.

[2] Karimi-Khouzani O, Heidarian E, Amini S A (2017). Anti-inflammatory and ameliorative effects of gallic acid on fluoxetine-induced oxidative stress and liver damage in rats. Pharmacol Rep.

[3] Lapchak P A, Zhang J H (2017). The high cost of stroke and stroke cytoprotection research. Transl Stroke Res.

[4] Boström P, Wu J, Jedrychowski M P, Korde A, Ye L, Lo J C (2012). A PGC1-a-dependent myokine that drives brown-fat-like development of white fat and thermogenesis. Nature.

[5] Young M F, Valaris S, Wrann C D (2019). A role for FNDC5/Irisin in the beneficial effects of exercise on the brain and in neurodegenerative diseases. Prog Cardiovasc Dis.

[6] Conti E, Grana D, Stefanoni G, et al (2019). Irisin and BDNF serum levels and behavioral disturbances in Alzheimer's disease. Neurol Sci.

[7] Zhao Y T, Wang J, Yano N, et al (2019). Irisin promotes cardiac progenitor cell-induced myocardial repair and functional improvement in infarcted heart. J Cell Physiol.

[8] Wu H, Guo P, Jin Z, et al (2019). Serum levels of irisin predict short-term outcomes in ischemic stroke. Cytokine.

[9] Li D J, Li Y J, Yuan H B, Qu L F, Wang P (2017). The novel exercise-induced hormone irisin protects against neuronal injury via activation of the Akt and ERK1/2 signaling pathways and contributes to the neuroprotection of physical exercise in cerebral ischemia. Metabolism.

[10] Bonita R, Beaglehole R (1988). Recovery of motor function after stroke. Stroke.

[11] Mehrabian S, Taheri E, Karkhaneh M, Qorbani M, Hosseini S (2016). Association of circulating irisin levels with normal weight obesity, glycemic and lipid profile. J Diabetes Metab Disord.

[12] Lee M J, Lee S A, Nam B Y, et al (2015). Irisin: A novel myokine is an independent predictor for sarcopenia and carotid atherosclerosis in dialysis patients. Atherosclerosis.

[13] Emanuele E, Minoretti P, Pareja-Galeano H, Sanchis-Gomar F, Garatachea N, Lucia A (2014). Serum irisin levels, precocious myocardial infarction, and healthy exceptional longevity. Am J Med.

[14] Jia J, Yu F, Wei W P, et al (2019). Relationship between circulating irisin levels and overweight/obesity: A meta-analysis. World J Clin Cases.

[15] Liu L, Zhang Q, Li M, et al (2021). Early post-stroke electroacupuncture promotes motor function recovery in post-ischemic rats by increasing the blood and brain irisin. Neuropsychiatr Dis Treat.

[16] Tu W J, Qiu H C, Cao J L, Liu Q, Zeng X W, Zhao J Z (2018). Decreased concentration of irisin is associated with poor functional outcome in ischemic stroke. Neurotherapeutics.

[17] Tu W J, Qiu H C, Liu Q, Li X, Zhao J Z, Zeng X (2018). Decreased level of irisin, a skeletal muscle cell-derived myokine, is associated with post-stroke depression in the ischemic stroke population. J Neuroinflammation.

